# Peste Des Petits Ruminants in the Middle East: Epidemiological Situation and Status of Control and Eradication Activities after the First Phase of the PPR Global Eradication Program (2017–2021)

**DOI:** 10.3390/ani13071196

**Published:** 2023-03-29

**Authors:** Camilla T. O. Benfield, Matteo Legnardi, Friederike Mayen, Ahmad Almajali, Giuseppina Cinardi, Dominik Wisser, Hassen Chaka, Felix Njeumi

**Affiliations:** 1 Food and Agriculture Organization of the United Nations (FAO), Viale delle Terme di Caracalla, 00153 Rome, Italy; 2 Dipartimento di Medicina Animale, Produzione e Salute (MAPS), Università di Padova, Viale dell’Università 16, 35020 Legnaro, Italy; 3 Regional Office for Near East and North Africa, Food and Agriculture Organization of the United Nations (FAO), Cairo P.O. Box 2223, Egypt; 4 Subregional Office for the Gulf Cooperation Council States and Yemen, Food and Agriculture Organization of the United Nations (FAO), Abu Dhabi 62072, United Arab Emirates; 5 Department of Veterinary Clinical Sciences, Faculty of Veterinary Medicine, Jordan University of Science and Technology, Irbid 22110, Jordan

**Keywords:** peste des petits ruminants, small ruminant morbillivirus, disease eradication, sheep, goats, small ruminant, wildlife, Middle East, PPR GEP

## Abstract

**Simple Summary:**

Peste des petits ruminants (PPR) is a viral disease of domestic and wild small ruminants. Due to its severe socioeconomic consequences for endemic countries across Africa, the Middle East and Asia, along with its threat posed to biodiversity, PPR is currently the target of a Global Eradication Program (PPR GEP), aimed at eradicating the disease by 2030. This review provides an overview of the progress of eradication activities throughout the Middle East, aiming to raise awareness and inform the next phases of PPR GEP in the region. PPR is either confirmed or suspected to be present in most of the fifteen considered countries, stretching from Egypt to the Islamic Republic of Iran. Some notable gaps are however apparent in surveillance capacity, hampering a full understanding of the epidemiological situation. Moreover, the vaccination campaigns and other control measures undertaken by many countries also appear insufficient in effectively reducing PPR incidence. To overcome these hindrances, the next phases of the PPR GEP should focus on the cooperation and coordination of control efforts, which are indispensable to achieve PPR freedom at the regional level.

**Abstract:**

Peste des petits ruminants (PPR) is a burdensome viral disease primarily affecting small ruminants, which is currently targeted for eradication by 2030 through the implementation of a Global Control and Eradication Strategy (PPR GCES). The PPR GCES, launched in 2015, has strongly encouraged countries to participate in Regional PPR Roadmaps, designated according to the Food and Agricultural Organization of the United Nations (FAO) and World Organisation for Animal Health (WOAH) regions and epidemiological considerations, with each targeted by dedicated meetings and activities. Following the conclusion of the first phase of the PPR Global Eradication Program (PPR GEP) (2017–2021), the present work focuses on the disease situation and status of the eradication campaign in the fourteen countries of the PPR GCES Middle Eastern Roadmap as well as Egypt. PPR is endemic to or suspected to be present in most of the region, except for Bahrain, which, as of 2021, is preparing to apply for official recognition as being free of PPR. Some substantial shortcomings are observed in surveillance and disease reporting, as well as in the implemented control strategies, most notably vaccination. Since many of these limitations are shared by many of the investigated countries, the international cooperation and harmonization of control efforts appears crucial to making PPR eradication attainable in the Middle East.

## 1. Introduction

Peste des petits ruminants (PPR) is a highly contagious viral disease that primarily affects small ruminants (SRs). First described in 1942 [1], the disease is currently endemic to large parts of Africa, Asia and the Middle East, endangering the social, economic and food security of dozens of low- and middle-income countries [2]. In addition to their burden on SR production, PPR outbreaks have also been documented in many wild ruminant species [3], posing a threat to biodiversity [4,5,6]. Camels may also develop clinical signs [7,8,9], while other domestic ruminants, for example, cattle and buffaloes, may become subclinically infected [10,11]. The role of wild and atypical hosts in PPR epidemiology is, however, poorly understood [3,5].

PPR is caused by the peste des petits ruminants virus (PPRV), an enveloped virus with a single-stranded, negative-sense RNA genome that belongs to the *Paramyxoviridae* family and *Morbillivirus* genus. Four PPRV lineages (I–IV), all belonging to a single serotype, can be discriminated based on their genetic differences. Lineage I circulation has been historically limited to West Africa; long thought to be extinct, it has recently been detected again in Mali [12]. Lineage II is present in West and Central Africa, while lineage III has been reported only in East Africa and in the southern part of the Arabian Peninsula [13]. Lastly, lineage IV, after a probable origin in West Africa, spread eastward and became endemic to large parts of Asia and the Middle East, but is now thought to have become the dominant lineage in Africa as well [4,14].

PPRV is closely related to rinderpest virus, a morbillivirus responsible for the disease of predominantly large ruminants, which in 2011, became the only animal disease to have been globally eradicated [15]. Building on the lessons learned from rinderpest eradication, the PPR Global Eradication Program (PPR GEP) was jointly launched by the Food and Agriculture Organization of the United Nations (FAO) and the World Organisation for Animal Health (WOAH, founded as the OIE) in 2016. To achieve PPR eradication, which is targeted for 2030, a PPR Global Control and Eradication Strategy (PPR GCES) was developed, based on a progressive reduction in PPR incidence and spread through targeted vaccination. PPR GCES relies on a four-stage stepwise approach: in Stage 1, countries are advised to focus on assessing the local epidemiological situation; Stage 2 is dedicated to controlling PPR infection, mainly through vaccination campaigns informed by surveillance; in Stage 3, eradication should be pursued by strengthening surveillance and preventive measures; lastly, in Stage 4, vaccination must be suspended and a body of evidence must be collected proving PPRV is no longer circulating [16].

To promote the cooperation and coordination of eradication efforts between neighboring countries, the PPR GCES established nine different regions, each one targeted by periodical Regional Roadmap meetings and overseen by a Regional Advisory Group (RAG). Following the conclusion of the first phase of PPR GEP (2017–2021), this review focuses on the epidemiological context and status of the eradication activities in the Middle Eastern Roadmap region and in the countries that are part of it. According to the original definition contained in the PPR GCES, this region encompasses fourteen countries, namely the Kingdom of Bahrain, the Islamic Republic of Iran, the Republic of Iraq, the Hashemite Kingdom of Jordan, the State of Kuwait, the Lebanese Republic, the Sultanate of Oman, Palestine, the State of Qatar, the Syrian Arab Republic, the Kingdom of Saudi Arabia (KSA), the United Arab Emirates (UAE), the Republic of Yemen and Israel (despite Israel belonging to the FAO and WOAH Regional Commission for Europe). Additionally, despite being part of the North African roadmap region, Egypt was considered in the present analyses. This decision was based on the epizone approach proposed in the PPR GCES, which supports the grouping of countries with shared epidemiological features even when included in separate roadmap regions [16]. In fact, Egypt has been actively involved in several of the meetings organized for Middle Eastern countries and has also been considered in the PPR Regional Strategy for the Middle East.

## 2. Materials and Methods

The data presented in this review were extracted from meetings and activities organized in the region during the first five years of the PPR GEP. The first Roadmap meeting for the Middle East was held in Doha (Qatar) in December 2015 and the second Roadmap meeting for the region was held in Amman, Jordan, in October 2017. Recent meetings analyzed for this review included: (i) a PPR GEP Regional Consultation on PPR control in the Middle East, held virtually on 31 March–1 April 2021 and hosted by the GF-TAD regional steering committee; (ii) regional training on the PPR PMAT, held virtually on 24–25 March 2021 and hosted by the Arab Organization for Agricultural Development (AOAD) in partnership with the FAO and WOAH; (iii) a PPR Control and Eradication Strategy follow-up meeting for the Gulf Cooperation Council (GCC) States and Yemen, held virtually on 1–3 March 2022 under the GF-TAD framework; and (iv) an FAO/WOAH Consultative Seminar on progress made in the FMD and PPR Regional Roadmap for East Mediterranean Countries, held on 11–13 September 2022 in Beirut, Lebanon (where, of the considered countries in this review, Lebanon, Egypt, Jordan, Iraq and the Syrian Arabic Republic presented). In these meetings, countries’ representatives presented updates on the PPR epidemiological situation and the status of PPR control and eradication activities at the national level. The outputs of plenary discussions were also considered, along with those of additional bilateral meetings between individual countries and the FAO. Other sources of information included the PPR Regional Strategy for the Middle East, drafted in 2016, and the results of self-assessments performed by countries using the revised version of the PPR Monitoring and Assessment Tool (PMAT), provided to countries prior to meetings. To complement the grey literature, additional data were retrieved from scientific publications. The official disease status and reported PPR outbreaks were taken from the WOAH WAHIS [17], while the FAOSTAT was consulted for SR population figures [18] and the data about SR population density were derived from the fourth iteration of the Gridded Livestock of the World [19]. Maps were prepared using QGIS 3.24 [20].

## 3. PPR Situation at the Regional Level

**Figure 1:**
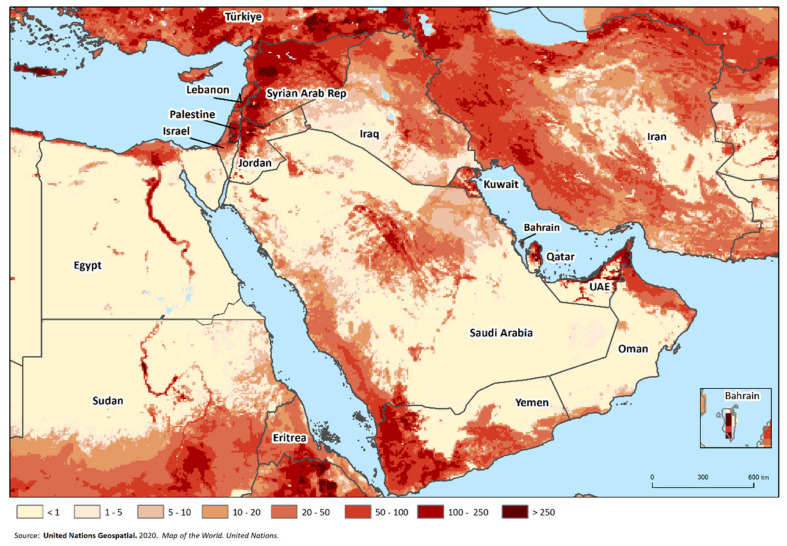
Map of the region showing the distribution of domestic SRs (sheep and goats), derived from GLW4 [19] and adjusted to FAOSTAT population figures for 2020 [18]. SR density (head per square km) is indicated by the colored shading.

The considered region spans approximately 6.5 million km^2^ and includes some of the countries that rely most heavily on SR production globally. More than 161 million SRs are reared in the Middle East (Table 1), representing 9.3% and 3.9% of the global sheep and goat populations [18]. Taken together, sheep and goats comprise more than 90% of the total national livestock population (excluding poultry) in multiple countries, including Bahrain, Jordan, Kuwait, Palestine, Saudi Arabia and Syria [18], and constitute the main source of income and food security for low-income smallholders. The density distribution of SRs is highly heterogenous across the region (Figure 1, Figures S1 and S2).

The SR production systems most frequently encountered throughout the region are traditional migratory or transhumance farming, along with semi-sedentary or sedentary forms of production [21,22,23,24,25,26]. The importance of the SR sector for the region is also testified by the trade volume of live sheep and goats. In 2020, the considered countries accounted for approximately 20% of the cumulative live sheep and goat exports and more importantly, for over 70% of the imports, with Saudi Arabia (26.7%), Kuwait (13.7%), Jordan (11.3%) and Qatar (7.5%) being the four largest importers in the world according to open-source data from the Observatory of Economic Complexity (OEC) [27] (Figure S3).

**Figure 2:**
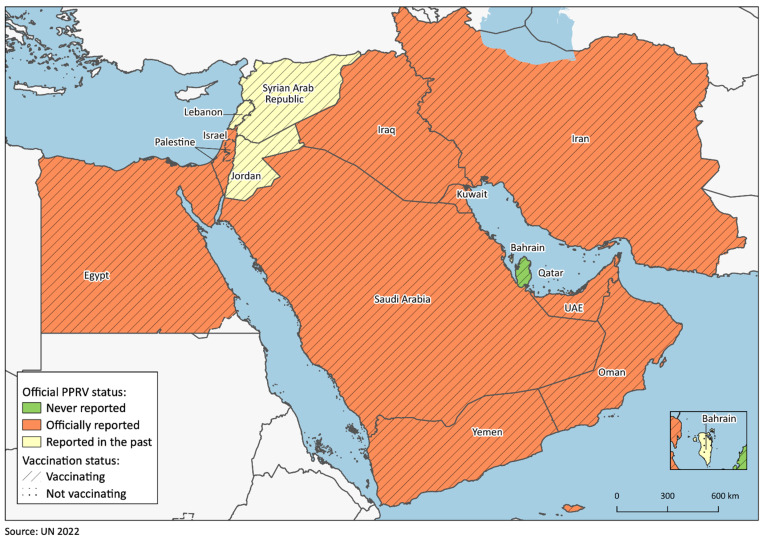
Map of the considered region showing each country’s PPR official status as reported in the WOAH WAHIS and current vaccination status.

Since sheep and goats constitute such a critical asset for the subsistence of a large part of the population, PPR represents a major threat for the economy and societies of the Middle East. According to a recent publication on global ecological niche modelling for PPR, only some of the countries belonging to the region, including Iraq, Iran and those bordering the Mediterranean Sea, represent highly suitable niches for PPR, while most of the Arabian Peninsula does not [28]. Nonetheless, since its first reports in the late 1970s [29], the disease has occurred in all 15 countries included in the present study. Nowadays, PPR is officially reported in 10 of the considered 15 countries, namely Egypt, the Islamic Republic of Iran, Iraq, Israel, Kuwait, Oman, Palestine, the KSA, the UAE and Yemen. Of the remaining countries, Bahrain last reported PPR in 2012, Jordan, in 2000 (but PPR is very likely present) and Lebanon, in 2005. Syria has not declared any cases in decades (although antigen and antibody positivity has been detected in the northwestern area), while PPR has never been reported in Qatar [17] (Figure 2). However, multiple studies support the actual presence of the disease in Qatar during the last decade [30,31]. Based on these data, and on the shortcomings hindering surveillance activities in multiple areas, the knowledge of the epidemiological scenario in the Middle East still appears incomplete, and PPR is likely present in more countries than those in which it is currently officially reported. Another factor indicating that PPR is also perceived as a tangible threat by all countries is that vaccination is administered in 14 countries of the region, albeit using different strategies. The sole exception is represented by Bahrain, where the import and sale of PPR vaccines is now illegal (Figure 2).

**Figure 3:**
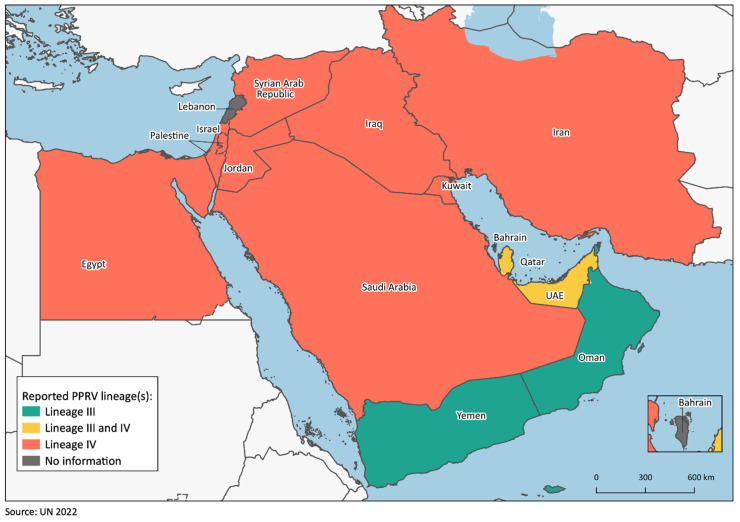
Map of the region showing all the PPRV lineages ever detected in each of the considered countries.

Standing at the junction between Africa and Asia, the Middle East is at the heart of the geographic range of PPR and abuts endemic countries at its western (Libya, Sudan), northern (Turkey) and eastern borders (Afghanistan, Pakistan). Evidencing its central role in PPRV spread between the two continents, this region is historically characterized by the circulation of both lineage III, which outside of the Middle East has been detected only in East Africa, and lineage IV, which, despite its estimated African origin, has been historically predominant in Asia [14]. The first detections of lineage III strains in the region occurred in Oman in 1978 and in the UAE in 1986 [32]. Based on a recent phylodynamic analysis, these two countries, together with Ethiopia, have been proposed as the possible place of origin of this lineage [14]. Nonetheless, this finding is in disagreement with an earlier study in which Benin was estimated as the country of origin [4], likely due to different sets of sequences being included in the two analyses. Lineage III was later recorded in Qatar in 2010 [30] and in Yemen in 2001 and 2013 [33,34]. On the other hand, lineage IV has been found in a larger number of countries. Since its first detection in Israel in 1993 [35], its presence has been evidenced in Egypt 2012 [36,37,38], Iran [39,40], Iraq [41], Jordan [42], Kuwait [33], Palestine [43], the KSA [30,44] and the UAE [45]. No PPR sequences are available for Bahrain and Lebanon. In the latter, the past circulation of lineage IV may be hypothesized based on the detection of Israeli strains in proximity to the border between the countries. In fact, the first PPRV incursion into Israel has been proposed to have occurred through Lebanon [35]. It is worth noting that the areas of detection of the two lineages tend not to overlap, except for in Qatar, where both lineages were found to circulate contemporaneously in 2010 [30], and in the UAE, where lineage III was reported in 1986 [32] and IV from 2009 onwards [45] (Figure 3). This information supports the existence of separate networks of PPR spread and maintenance in the Middle East, although more molecular epidemiological data would be needed to substantiate this hypothesis. Moreover, it remains possible that lineage IV is also present in Oman and Yemen, although not yet identified (Figure 3).

**Figure 4:**
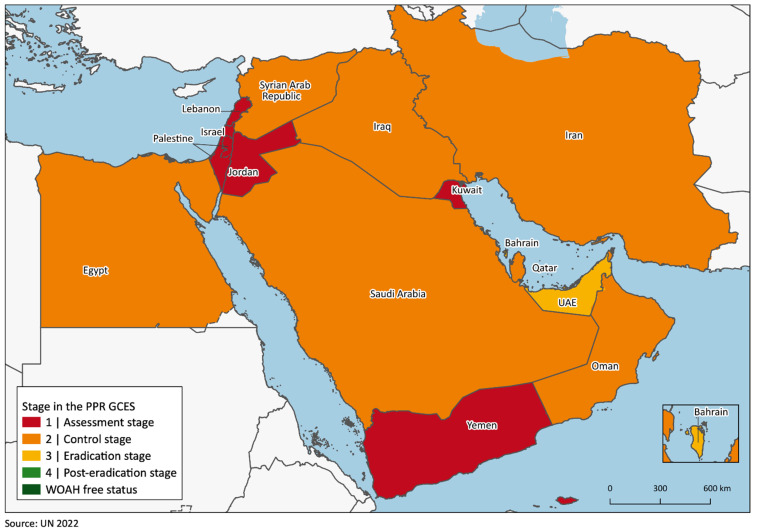
Map of the region detailing each country’s stage within the stepwise approach of the PPR GCES according to the latest self-assessment data (2017 for Iraq; 2021 for Egypt, the Islamic Republic of Iran, Israel, Kuwait, Lebanon, Syria; 2022 for Bahrain, Jordan, Oman, Palestine, Qatar, KSA, UAE, Yemen).

When considering the surveillance and control activities enacted against PPR, the regional situation appears quite uniform, as testified by the individual countries’ positions within the PPR GCES’s stepwise approach, with thirteen out of fifteen countries placed in one of the first two stages according to self-assessment data. Specifically, Israel, Jordan, Kuwait, Lebanon, Palestine and Yemen are in Stage 1, while Egypt, the Islamic Republic of Iran, Iraq, Oman, Qatar, the KSA and the Syrian Arab Republic are in Stage 2. The two remaining countries, Bahrain and the UAE, are placed in Stage 3 (Figure 4; Table S1).

To promote international cooperation and advance PPR eradication in a harmonized manner, a PPR Regional Strategy for the Middle East was formulated in 2016, involving all the countries considered in this review except for the Islamic Republic of Iran, Israel and Palestine. The Regional Strategy provided an overview of the epidemiological context, identified the strengths and needs at the national and international levels and formulated a roadmap towards regional PPR eradication. Among the main measures proposed in the Regional Strategy was the establishment of a Regional Advisory Group (RAG) to oversee PPR control activities in the Middle East, as foreseen in the PPR GCES. The RAG comprises three Chief Veterinary Officers and the leaders of the Epidemiology Network and the Laboratory Network (as voting members), while regional FAO and WOAH representatives, representatives of the Regional Economic Communities and the PPR Secretariat participate in RAG meetings as non-voting members.

To promote coordination, another proposal contained in the Regional Strategy is the division of the Middle East into three groups of countries based not only on geographical proximity, but also on shared epidemiological and economic conditions and the availability of resources required for PPR control. One of these groups was proposed to be composed of Lebanon, the Syrian Arab Republic, Jordan and Iraq, with Jordan acting as the lead in light of its greater socioeconomic stability and the presence of diagnostic- and vaccine-producing facilities. Another proposed group was composed of members of the Gulf Cooperation Council (GCC), namely Bahrain, Qatar, Oman, Kuwait, the KSA and the UAE (fairly homogeneous in terms of economic prosperity and diagnostic resources), as well as Yemen based on geographic vicinity. For this subgroup of countries, Kuwait was proposed to coordinate laboratory activities, while the KSA was intended to ensure vaccine production. Lastly, Egypt was seen as a standalone country due to its location, size and internal availability of all the necessary infrastructures and could offer its support to neighboring PPR-endemic countries which were not included in the Regional Strategy, such as Sudan, Libya and Palestine. However, there is a clear need to review and update the regional strategy, in line with the Blueprint for the Second and Third Phases of the PPR GEP, launched in November 2022 [46] and its focus on identifying and targeting key episystems, i.e., interconnected host populations capable of maintaining virus circulation and transmission, which are often transboundary in nature.

Despite the harmonization efforts undertaken in the region, many differences persist at the individual country level in terms of the understandings of PPR epidemiology and burden, preparedness of veterinary services, available funding, legal frameworks and implemented surveillance and control activities, which are reviewed in detail in the following section.

## 4. PPR Situation at the National Level

### 4.1. Bahrain

PPR was last recorded in Bahrain in 2012, with 219 cases and 25 mortalities reported in sheep imported from Africa between January and June [47].

According to the PMAT self-evaluation conducted in 2022, the available diagnostic capacity includes ELISA assays and RT-PCR, but not sequencing. Passive surveillance is conducted and a disease reporting system is in place but still needs to be digitized. Vaccination against PPR is currently prohibited by law in order to progress towards official recognition of being PPR-free. However, Bahrain still imports livestock from countries where vaccines are used and PPR is endemic, including other Middle Eastern countries such as Oman, Lebanon and Jordan. As a result, as of 2021, SRs were still found serologically positive.

According to the last assessment in 2022, Bahrain is at the third stage of the stepwise approach and is currently in the process of preparing a contingency plan and dossier to apply to the WOAH for recognition of official PPR-free status. To support these efforts, along with the elimination of other animal diseases and zoonoses and the conservation of animal genetic resources, the FAO has donated USD 1.3 million under the project UTF/BAH/006/BAH, which covers the period from 2019 to 2022.

### 4.2. Egypt

**Figure 5:**
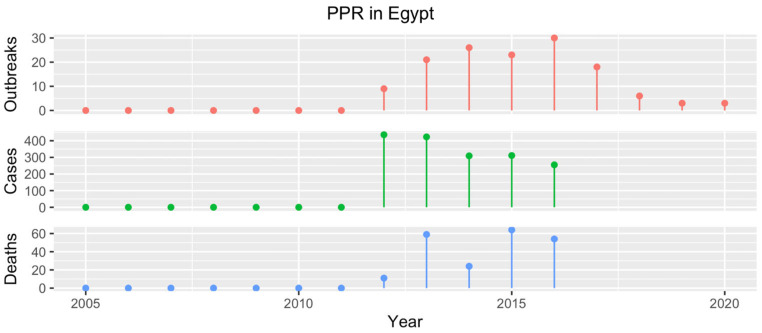
Number of PPR outbreaks (red), cases (green) and resulting deaths (blue) reported in Egypt from 2005 to 2020. Data from 2005 to 2016 were obtained from the WOAH WAHIS [17], while the number of PPR outbreaks occurring in the years 2017–2020 was communicated directly by country representatives at consultation meetings.

The first PPR cases were documented in Egypt in 1987 in goats from the Giza Governorate, and in 1989, in lambs from the Faiyum Governorate [48,49]. From 1989 to 2012, PPR was never officially recorded in the country, although its ongoing circulation was evidenced by the results of several serological surveys [50,51,52]. Historical studies have also reported PPRV seropositivity in camels, although they require cautious interpretation [7,53]. In 2012, PPR outbreaks were documented in sheep flocks in the Ismailia and Cairo Governorates [17,36], leading to the development and application of a national strategy to control the disease. Subsequently, PPR has been reported annually (Figure 5) and multiple studies have found considerable seroprevalence rates in unvaccinated SRs sampled in different governorates [37,38,54,55,56].

All the strains ever characterized from Egypt belong to lineage IV [36,37,38]. A high homology with Sudanese viruses was noted, which, along with the serological evidence of PPR infection in SRs [57] and cattle [58] imported or smuggled from Sudan, supports the key role of Egypt in the eastward spread of PPR from Africa to the Middle East as suggested by several studies [30,36,58].

PPR control and eradication activities are currently conducted within the framework of an NSP endorsed in 2018 and covering 5 years. The available diagnostic assays include Ab ELISA and RT-PCR and are performed by the Animal Health Research Institute (AHRI). Both passive and active surveillance are carried out. The latest data come from a field investigation performed between August 2020 and January 2021, in which 281,688 SRs from 4602 villages were clinically examined without finding any suspected PPR cases.

Vaccination is funded by the government and employs a Nig 75/1-based live vaccine locally produced by the Veterinary Serum and Vaccines Research Institute (VSVRI). Every batch is independently tested and certified by the Central Laboratory for the Evaluation of Veterinary Biologics (CLEVB). Mass vaccination has been conducted since 2017, aiming to immunize all SRs for 3 years and then only newborns for another 2 years. However, the vaccination coverage reached in 2017–2020 was always between 30 and 35%, except for in 2018 when only 265,041 doses were administered. Ring vaccination is also undertaken within a 2 km radius around suspected outbreaks. A post-vaccination monitoring (PVM) strategy for 2021, but not the results, were reported by country representatives.

PPR control in Egypt is reportedly hampered by the incomplete identification and registration of SR and the difficulty in regulating the movements of nomadic flocks. However, studies to understand the drivers of SR mobility are ongoing (as of September 2022), supported by EU-FMD in collaboration with CIRAD. A Community-based Animal Health Outreach (CAHO) program, aimed at improving disease detection and reporting through participatory epidemiology, was initiated in the country in 2015 with the support of the FAO and the United States Agency for International Development (USAID), but the engagement of farmers and private veterinarians in PPR-related surveillance and control is reportedly still limited. Based on a self-evaluation in 2021, Egypt is at the second stage of the stepwise approach.

### 4.3. Islamic Republic of Iran

Based on an epizone approach and Iran’s participation in Roadmap meetings organized in the Economic Cooperation Organization (ECO) region, the PPR situation in the country, which is currently placed at the second stage of the stepwise approach, has already been reviewed in a previous analysis focusing on Central and Eastern Asia and West Eurasia. For further information, see Legnardi et al. [59].

### 4.4. Iraq

The first evidence of PPR presence in Iraq came from a serological survey performed in 1997, which found a seroprevalence rate of 21.6% and 30.9% in sheep herds from the central and northern regions, respectively [60]. The first officially reported outbreak occurred the following year, in September 1998, in the Nineveh Governorate in northern Iraq [61]. Local authorities responded to this outbreak by vaccinating 250,000 SRs in the area with a rinderpest vaccine and requesting the FAO’s assistance to receive a provision of PPR vaccines and build diagnostic and surveillance capacity [62].

In recent years, annual compulsory vaccination campaigns have been conducted free-of-charge towards farmers of all SRs older than three months. Between 2011 and 2018 (with the exception of 2015, for which no information is available), the reported annual vaccination coverage rates were as high as 91% (2013) and as low as 67.8% (2018) [63]. Despite these efforts, PPR outbreaks kept being observed with no significant improvements [41,63,64,65], and the continuing circulation of the virus has been attested by several serological studies conducted in different parts of the country [66,67,68,69]. In addition, PPR was responsible for more than 750 deaths among wild goats (*Capra aegragus*), whose conservation status is currently considered as near-threatened according to the IUCN [70], documented in the Iraqi Kurdistan region in 2010–11 [65,71]. Moreover, a laboratory-confirmed PPR case in a deer in 2021 and seropositivity in cattle (22.3%) and buffalos (22%) sampled across 15 governorates were also reported at the 2022 consultation meeting (caveated by the lack of validated diagnostic tests for these species). It may therefore be important to consider atypical hosts for PPR epidemiology or as indicator species to monitor the epidemiological situation, especially since joint breeding activities occur between SRs and cattle and buffalos in Iraq.

Among the reported constraints hindering PPR control are the impossibility of delivering veterinary services to insecure areas, uncontrolled animal movements across national borders, low cooperation and awareness among livestock owners and the veterinary private sector and a lack of diagnostic kits and appropriate training of veterinary personnel. In light of these findings, Iraq is currently placed at the second stage of the stepwise approach as of 2017.

### 4.5. Israel

**Figure 6:**
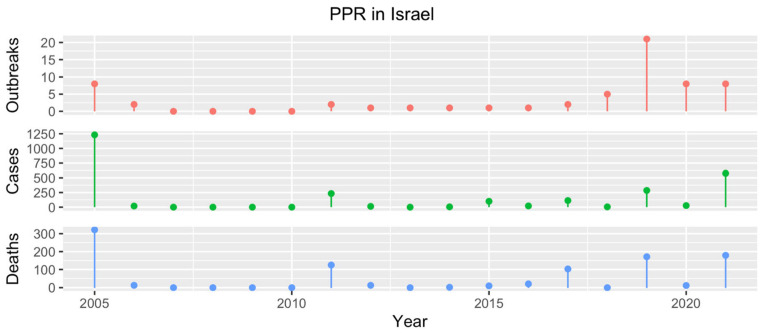
Number of PPR outbreaks (red), cases (green) and resulting deaths (blue) reported in Israel from 2005 to 2021 according to official reports available in the WOAH WAHIS [17].

The first documented outbreak of PPR in Israel occurred in 1993 [72] and represented the first detection of a PPRV belonging to lineage IV in the region [32]. Since then, PPR has been reported intermittently, causing more than 50 outbreaks from 2005 and 2021 [17] (Figure 6).

A phylogenetic study considering 18 full genome sequences collected in Israel from 1997 to 2014 revealed that all strains belonged to a single clade within lineage IV [35], supporting the occurrence of a single PPR introduction, supposedly from Lebanon [32]. The evolution of PPRV appears therefore segregated from the rest of the Middle East, fitting with the sociopolitical peculiarities of the country compared to the rest of the region. The only exception was represented by the close similarity between Israeli and Palestinian strains, which may be explained by the past occurrence of uncontrolled animal movements across the border [35]. In December 2016, PPR was reported in the Nubian ibex (Capra nubiana) [73]. Although the affected herd was kept in captivity, this finding highlights the potential threat posed by the disease to indigenous wild SR species [74].

A compulsory vaccination program was conducted annually for all SRs older than 3 months of age [32] until 2016, when it was discontinued and vaccination was made non-mandatory. No data on vaccination coverage or seroconversion are therefore available since that date. Despite not participating in regional PPR meetings and apparently never conducting a self-evaluation using the PMAT, Israel is at the first stage of the stepwise approach, as self-reported in 2021.

### 4.6. Jordan

The first evidence of PPR in Jordan comes from a serological survey performed in 1990 [75]. In 1993, a strain belonging to lineage IV was recognized as responsible for a single outbreak [42,76]. More than a decade later, a serological survey conducted between 2006 and 2007 in five governorates in the northern regions found 60% and 74% seroprevalence rates in sheep and goat flocks, respectively [42]. Since 2000, however, there are no official records of PPR outbreaks [17], although the disease still occurs in the country. The reasons for this lack of reporting may include the low awareness and compliance among farmers and of an enabling legal framework. PPR diagnosis is only performed at the central laboratory, and the diagnostic capacity includes ELISA and RT-PCR but no gene sequencing, although this is reportedly severely limited by issues such as understaffing, a lack of training in field veterinarians for proper sample collection and handling and the absence of quality assurance and control protocols. Surveillance relied on a paper-based reporting system until 2022, when an Electronic Integrated Disease Surveillance System (EIDSS) was introduced in four governorates, with plans to extend it to the rest of the country by the end of 2023. An animal identification system using plastic ear tags has been in use since 2018, but livestock movements are not monitored and no restrictions are enforced in the case of PPR outbreaks. A lack of epidemiological assessment of PPR in the country is a major recognized gap, although plans for serosurveillance became underway in 2022. The population of camels in the country should also not be overlooked for PPR surveillance. No surveillance is conducted of wildlife, although the chances for wild and domestic SRs to come into contact are reportedly limited since wildlife is only present in protected areas.

Vaccination is carried out annually with a Nig 75/1-based vaccine locally produced by JOVAC. The coverage rates reached in recent years are listed in Table 2.

Although not performed hitherto, there is the intention to perform mass vaccination against PPR in the future, likely coordinated with FMD vaccination. PVM activities have not yet been performed. In 2016, a serosurveillance study funded by the FAO in cooperation with the Jordan University of Science and Technology (JUST) found a 49% positivity in sheep and 50% in goats, but vaccination status was not taken into account and hence, these data cannot be used to inform as to the effectiveness of the vaccination campaign.

Although identified as the animal disease with the second highest priority after FMD, PPR is not yet covered by an NSP. However, in summer 2022, a PPR coordination committee was established and performed a PMAT self-evaluation. Thanks to multiple assessments performed throughout the years, including a WOAH Performance of Veterinary Services (PVS) Gap Analysis mission in 2017 and multiple PMAT self-evaluations, the weaknesses of the national veterinary services and PPR control and eradication activities are well-known. To address them, Jordan has expressed the need for support from international organizations in terms of funding, training activities, value chain analysis and assistance to develop its PPR-specific legislation and NSP, which is ongoing. Based on the current conditions, Jordan is placed at the first stage of the stepwise approach.

### 4.7. Kuwait

**Figure 7:**
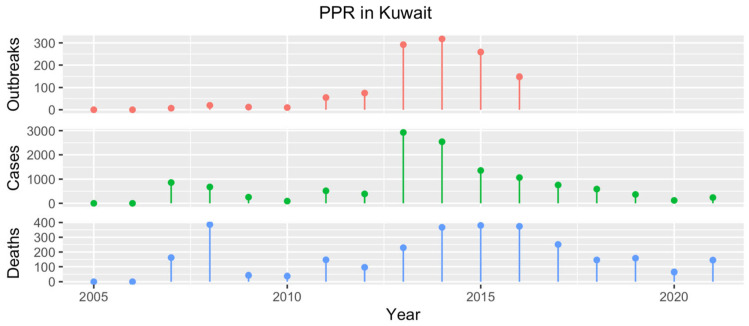
Number of PPR outbreaks (red), cases (green) and resulting deaths (blue) reported in Kuwait from 2005 to 2021. Data from 2005 to 2016 were obtained from the WOAH WAHIS [17], while the information regarding PPR cases and related deaths occurring in the years 2017–2021 were presented by a national representative during the 2022 meeting for GCC countries and Yemen.

The first PPR outbreak in Kuwait occurred in 1989. After going unreported from 1991 to 2007, the disease was then notified every year, peaking around 2013–2015 (Figure 7). All the outbreaks were reported in domestic SRs, except for one observed in November 2007 in the Al Farwaniyah Governorate, which was responsible for 20 cases and 13 deaths among wild SRs (species unidentified) [17]. The only molecular characterization performed to date was for a strain in 1999, shown to belong to lineage IV [33].

Diagnostic activities, including agar gel immune-diffusion (AGID), Ab and Ag ELISAs and RT-PCR, are conducted at the Central Veterinary Diagnostic Laboratory, but are reportedly hindered by a lack of funding along with the need to improve laboratory quality assurance mechanisms. The enacted passive surveillance strategy, carried out by the epidemiology and zoonosis unit of the Animal Health Department, also needs to be enhanced and its information system should be digitized to ensure a faster response to suspected cases.

A national control program is in place and PPR is a notifiable disease, but an NSP has yet to be drafted and a National PPR Committee has not yet been established. Vaccination was carried out in 2019 (217,318 doses, 22.8% coverage) and in 2020 (629,916 doses, 67.0% coverage) and is performed in response to outbreaks, along with movement restrictions. However, a lack of compliance has been reported among livestock keepers, whose cooperation is reportedly limited by the absence of legislated compensation mechanisms. As a result, the disease is underreported and emergency vaccination is difficult to administer. Currently, Kuwait is at the first stage of the stepwise approach.

### 4.8. Lebanon

PPR was reported in Lebanon in 1978 [77] and throughout the 1990s [78,79]. An epidemiological survey conducted in 2004–2006 across all districts revealed the presence of serologically positive animals in 75.8% of the investigated sheep flocks, 62.5% of the goat flocks and 13.5% of the cattle farms, despite vaccination not being administered [80]. Similarly, Attieh et al. [79] reported an 89.4% seroprevalence among 47 goat flocks of unknown vaccination status sampled from all regions during 2005. Although these findings support extensive PPR circulation in the country at the time, the disease has never been officially reported from 2005 onwards [17]. Nonetheless, problems related to staff shortages, underreporting and underinvestigation have been reported to have occurred at the time.

A nationwide mass vaccination campaign was conducted from 2010 to 2016. PVM was last carried out in 2017, revealing a reported 40–92% and 70–95% seropositivity rate in sheep and goat flocks, respectively. Vaccination and PVM were both carried out with the support of the FAO and UK Aid. From 2017 onwards, the Ministry of Agriculture has been severely underfunded due to currency devaluation, the COVID-19 pandemic and the Beirut port explosion, resulting in significant gaps in terms of surveillance, prevention and control. In 2019, the veterinary services benefited from an EU twinning project entitled “Strengthening the Veterinary Services and Food Safety Capacities of the Lebanese Ministry of Agriculture” (LB 15 ENI AG 03 18) [81]. A total amount of EUR 1.85 million was donated to help secure the health and safety of the national animal production and food industry. In addition, the World Bank through the FAO provided 600,000 doses of PPR vaccines which were used between March and August 2022.

Currently, a proper surveillance system is being reestablished in the country, relying on the diagnostic capacity of the Lebanese Agricultural Research Institute (LARI), which includes Ab ELISA and RT-PCR. Nonetheless, the PPR epidemiological situation remains obscure and Lebanon is at the first stage of the stepwise approach according to the self-assessment performed in 2021.

### 4.9. Oman

**Figure 8:**
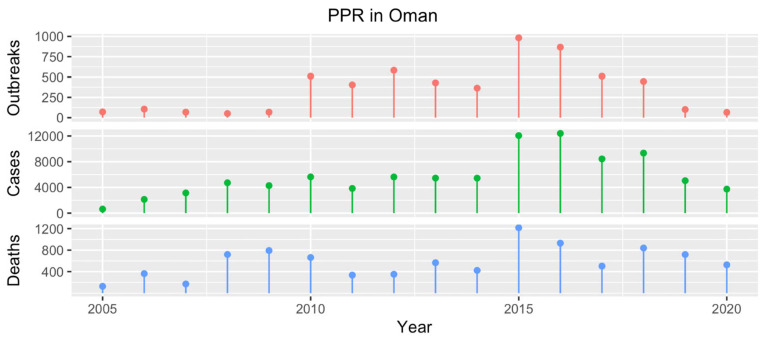
Number of PPR outbreaks (red), cases (green) and resulting deaths (blue) reported in Oman from 2005 to 2020. Data from 2005 to the first semester of 2017 were obtained from the WOAH WAHIS [17], while the information for the years 2018–2020 were presented by a national representative during the 2021 PPR GEP Regional Consultation on PPR control in the Middle East.

The first report of PPR presence in Oman dates back to 1978 [29]. In 1983, strains belonging to lineage III were detected both in goats [82] and wildlife [30]. Over the years, PPR has continuously represented a severe burden to the national SR sector, with over 5600 outbreaks registered between 2005 and 2020 (Figure 8).

Diagnostic capacity includes both ELISA and RT-PCR (without sequencing), which are used to conduct passive surveillance. The control of PPR, along with other infectious diseases including FMD, sheep and goat pox and rabies, is pursued through a National Livestock Vaccination Project carried out annually by 25 dedicated teams with the support of additional staff from the 70 governmental veterinary clinics. The reported number of SRs vaccinated against PPR was 964,227 in 2018 (33.2% coverage, according to SR population figures retrieved from the FAOSTAT), 913,364 in 2019 (30.8% coverage) and 1,629,576 in 2020 (53.9% coverage), far higher than the figures reported for 2011 (249,460, 11.6% coverage) and 2012 (277,009, 12.6% coverage) [18], but still insufficient in effectively pursuing eradication. To prevent further PPR introduction, the import of SRs is permitted only from PPR-free countries, or if the animals are kept for at least 21 days prior to their entry in an establishment with no history of PPR and located in a non-infected zone, or if they spent the previous 21 days in a quarantine station and were vaccinated before that time. Despite these measures, however, the number of PPR outbreaks remains high, although it is seemingly on a downward trend in recent years (Figure 8). According to the latest self-assessment in 2022, Oman is at the second stage of the stepwise approach.

### 4.10. Palestine

**Figure 9:**
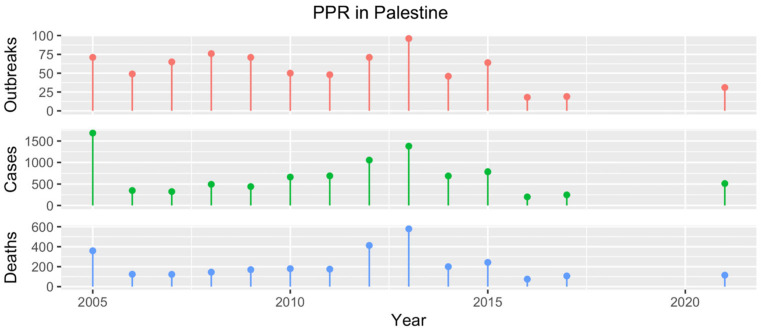
Number of PPR outbreaks (red), cases (green) and resulting deaths (blue) reported in Palestine from 2005 to 2021. Data from 2005 to 2017 were obtained from the WOAH WAHIS [17]. For 2006 and 2017, the available information covered only the first semester. No figures were available for the years 2018–2020, while those recorded in 2021 were communicated directly by country representatives.

PPR is endemic in Palestine, with over 850 outbreaks reported between 2005 and 2017 [17,43] (Figure 9). However, the actual number is likely far higher, as the reported understaffing and lack of governmental support only allows for limited outbreak investigation activities. In particular, little is known regarding PPR occurrence within the West Bank and Gaza Strip, where disease reporting relies on poorly defined and inefficient communication channels. In particular, private veterinarians usually report diseases to the district veterinary office only when treatment is inconclusive. From the district offices, the information reaches the central veterinary services only through monthly disease bulletins or indirectly through the diagnostic reports sent by the Central Veterinary Laboratory in Hebron, where PPR diagnosis is performed. This mechanism often results in an incomplete, untimely knowledge of PPR outbreaks, hampering subsequent control activities.

PPR surveillance and control activities are conducted within the framework of an NSP drafted in 2019. PPR diagnosis is by RT-PCR, while serology is not available. An Animal Identification and Registration System is also in place, but in 2017, it was estimated that only 60–80% of the SR population was properly identified, with large differences between districts due to the limited compliance of livestock owners, making it difficult to prevent uncontrolled movements and animal smuggling. For the same reasons, quarantine of affected herds is envisioned but hard to enforce. No compensation is provided in the case of PPR outbreaks.

One of the biggest obstacles to PPR control in the past in Palestine has been the low coverage achieved by the vaccination program, which has historically been hampered by vaccine shortages and logistical issues caused by the sociopolitical situation [43]. Between 2005 and 2014, annual coverage was never higher than 22% and as low as 4% [43]. In that period, vaccines were administered based upon owners’ demand or in response to outbreaks. However, thanks to the FAO’s donations of 0.9 million vaccine doses in both 2021 and 2022, the Palestinian veterinary services were able to organize a nation-wide, free-of-charge vaccination campaign, achieving an immunization coverage of 80% at the country level in 2021. In 2022, the FAO provided country-tailored training to frontline veterinarians to support the vaccination and planned post-vaccination seromonitoring campaigns that year. During the vaccination campaigns, leaflets were distributed to farmers to raise awareness about PPR. In February 2022, an additional vaccine stock of 200,000 doses, procured through the Ministry of Agriculture, was administered during a non-mandatory campaign partially covered by the owners. Importers and traders of SRs are also requested to vaccinate their animals at their expense during quarantine and before sale.

Currently, Palestine is at the first stage of the stepwise approach.

### 4.11. Qatar

PPR has never officially been reported in Qatar. However, several studies suggest the presence of PPR in the country. The first was in 2010, when viruses belonging to lineages III and IV were identified in goats [30]. In another molecular survey conducted on retrospective samples taken between 2009 and 2016 from clinically suspected outbreaks, 51% of the tested animals were found positive by real-time RT-PCR and confirmed by viral isolation and Ag ELISA. Among the positive animals were sheep, goats and wild SRs including oryx (*Oryx leucoryx*), addax (*Addax nasomaculatus*), deer, gazelles, springbuck (*Antidorcas marsupialis*), waterbuck (*Kobus ellipsiprymnus*) and blackbuck (*Antilope cervicapra*) [31]. In addition, during the 2022 meeting for GCC countries and Yemen, Qatari representatives presented the results of a serological survey conducted on suspected clinical samples between 2017 and 2021, which showed significant seropositivity in oryx and gazelles (although these data should be caveated by the fact that serological diagnostic assays are not yet validated in wildlife species).

The national epidemiological situation appears therefore far from being understood. In light of these findings, a more rigorous surveillance activity is currently being planned in Qatar. The present diagnostic capacity includes Ab and Ag ELISAs and RT-PCR (although sequencing is not currently carried out) but is reportedly hampered by the lack of a continuous supply of diagnostic kits.

To ensure the prompt investigation of the disease, any suspected case should be reported to the competent authorities. An animal identification system has also been in use since 2004 in sheep, goats, cattle and camels. Yearly vaccination of all sheep and goats older than three months is mandatory, although no PVM is conducted. The number of vaccinated SRs and coverage rates reached in the last years are provided in Table 3.

Unfortunately, control efforts are reported to be hindered by the lack of compliance among livestock owners, who often refuse to vaccinate their animals, do not follow movement restrictions and fail to report suspected cases. According to the latest PMAT self-evaluation conducted in 2022, Qatar is currently placed at the second stage of the stepwise approach.

### 4.12. Kingdom of Saudi Arabia

PPRV was first isolated in the eastern part of the KSA in 1988 [83]. However, its presence has been suspected since the late 1970s based on clinical and serological findings, first in sheep [84] and then in deer and gazelles [85]. During the 1990s, a low number of outbreaks was documented [86], but since then, PPRV has become endemic in the country, as testified by several studies over the years, which reported PPR outbreaks both in domestic SRs [44,54,87,88,89] and in wild animals kept under semi-free-range conditions [90,91]. All PPRV strains ever characterized in the KSA belong to lineage IV [44,92,93]. In addition, multiple surveys reported variable seroprevalence rates in unvaccinated SRs from different parts of the country [88,94,95,96,97,98], including camels [44].

Diagnostic activities are carried out by the Central Veterinary Diagnostic Laboratory along with five regional laboratories, with the capacity to perform Ab and Ag ELISAs, viral isolation, RT-PCR and PPRV genome sequencing. According to the endorsed PPR Control Plan, serological passive surveillance is regularly conducted in domestic SRs, although it was reported that integration between the surveillance systems of the Ministry of Environment, Water and Agriculture (MEWA) and the Saudi Wildlife Authority is needed to effectively capture PPR events occurring in wild animals.

Vaccination is conducted by using a Nig 75/1-based vaccine locally produced by the MEWA, and herd immunity and vaccination coverage are assessed annually. Response actions to PPR outbreaks include ring vaccination, animal movement restrictions and active surveillance. However, no provisions are reportedly available for emergency funding.

Among the main constraints reported during the 2021 Roadmap meeting, country representatives stated that approximately 40% of sheep and goat herds were nomadic and located in remote areas, hampering vaccination activities. A lack of awareness among traditional livestock keepers on the importance of vaccination, disease reporting and animal identification has also been noted. Difficulties are also encountered in border control, which is especially relevant since the KSA imports millions of heads each year, especially from East Africa and during the Hajj pilgrimage season. For instance, Mahmoud and Galbat [89] documented a clinical outbreak in an unvaccinated sheep flock imported from Uruguay in 2012, which after entrance and coming in contact with local herds, quickly become infected with both PPRV and food-and-mouth disease virus (FMDV). The KSA has a major role in global sheep and goat trade and in 2020 was the top importer, with a share of 26.7% of global SR imports valued at USD 391 million [27].

PPR-related activities in the KSA recently benefited from an FAO donation under the project UTF/SAU/051/SAU, running from 2019 to 2025 and amounting to USD 93.3 million, whose aim is to strengthen the MEWA’s capacity to implement the Sustainable Rural Agricultural Development Program. As of 2022, the KSA is placed at the second stage of the PPR GCES stepwise approach.

### 4.13. Syrian Arab Republic

PPR is a notifiable disease by law in the Syrian Arab Republic, but is not currently reported, with no confirmed cases in decades. Notwithstanding this, in opposition-held parts of northwestern Syria, PPR outbreaks in goats were identified by rapid field antigen tests in 2016 and, in the same area, a survey coordinated by the FAO Cross-Border Unit for northwestern Syria in 2019 showed 35% PPR seropositivity in the absence of any prior vaccination.

Country representatives have reported that active and passive surveillance are carried out at the national level. Between 500 and 1000 sera were investigated by ELISA yearly between 2016–2020, and all suspected cases were investigated by RT-PCR (although gene sequencing is not available) without finding any positive result. However, syndromic surveillance is reportedly weak in the country.

A mass vaccination campaign was conducted in 2020 with the support of the FAO with a Nig 75/1-based vaccine supplied by JOVAC (Jordan). More than 10 million doses were administered at the national level, giving 85% vaccination coverage. Vaccination was also carried out in 2021, covering only newborns with the administration of 3 million doses at the farmers’ expense. Another 3 million doses were intended for administration in 2022, although according to a presentation from the country in September 2022, only 947,793 SRs had been vaccinated to date. Vaccination is performed by public veterinarians, with the support of veterinary associations. Apparently, samples were collected from vaccinated animals in 2022 for PVM but the data were not presented.

An early warning and emergency system is in place within the scope of an NSP which was officially adopted in 2019. The total cost of the NSP, which covered three years, was set at USD 928,950, of which USD 34,450 came from the national budget.

Despite the reported efforts, more data would be needed to properly evaluate the adequacy of the conducted surveillance and control activities along with the PPR epidemiological situation in Syria, whose PPR understanding and veterinary services capacity is hampered by the lasting political unrest and the resulting economic and logistic issues. Among the main constraints raised by national representatives reported are the shortages of PPR vaccines and diagnostic kits (Ab ELISAs in particular), the difficulties to control internal and transboundary animal movements and the need for capacity-building for technical staff. Considering these constraints, the Syrian Arab Republic is currently placed at the second stage of the stepwise approach according to the country’s presentation at the 2021 PPR consultation meeting.

### 4.14. United Arab Emirates

Ever since its first detection in 1986 [99], PPR has been endemic in the UAE. The disease was reported until 2010 and then from 2016 onwards, not only in domestic SRs but also in wild species [17]. For instance, Kinne et al. [45] found PPR to be responsible for two clinical outbreaks occurring in wildlife kept under semi-free-range conditions, one in winter 2005–2006 and another in winter 2008–2009. Among the diseased animals were different species of gazelles (Rheem gazelle, *Gazella subgutturosa maric*; Arabian mountain gazelle, *Gazella gazella cora*; springbuck, *Antidorcas marsupialis*; Arabian gazelle, *Gazella gazella*) and antelopes (bushbuck, *Tragelaphus scriptus*; impala, *Aepyceros melampus*), along with the Nubian ibex (*Capra nubiana*), wild Barbary sheep (*Ammotragus lervia*) and Afghan Markhor goat (*Capra falconeri*). The PPRV strain detected in 1986 belonged to lineage III [32], while only lineage IV strains were found during more recent sequencing activities conducted by Kinne et al. [45], the Pirbright Institute in 2014 and the Abu Dhabi Agriculture and Food Safety Authority in 2021.

The national diagnostic activities involve different institutions, including ADAFSA, the Central Veterinary Research Laboratory (CVRL), the Ministry of Climate Change and Environment (MOCCAE) and the Dubai Municipality (DM). The available techniques comprise viral isolation, Ab and Ag ELISAs, histopathology, RT-PCR and real-time RT-PCR. Since 2021, gene sequencing has also been available. All labs are reportedly ISO-accredited and approved by the United Kingdom Accreditation Service (UKAS) and routinely participate in laboratory proficiency testing programs organized at the national level.

Based on a presentation delivered at the 2021 Roadmap meeting, passive surveillance is conducted clinically and by Ag ELISA, with clinical surveillance and a rapid alert and response system operating for both domestic and wild animals as part of the National Biosecurity Notification System implemented in 2019. Active surveillance is not currently undertaken but is planned for future stages of the eradication process when vaccination is ceased.

The national PPR control and eradication plan relies on a mass vaccination strategy, which was adopted in 2016 in the context of a broader Animal Health Plan [100]. Annual vaccination campaigns are conducted in sheep, goats and captive antelopes and target all animals older than 3 months, which are then revaccinated every three years. The live vaccine used is based on a strain of Nig 75/1 and is mainly supplied by JOVAC (Jordan). The numbers of sheep and goats vaccinated in the last years were 1,612,491 in 2018 (36.2% coverage, according to SR population figures reported in the FAOSTAT), 2,386,806 in 2019 (52.5% coverage), 2,033,830 in 2020 (46.2% coverage) and 2,607,703 in 2021 (59.4% coverage) [18], not including vaccination activities conducted by the private sector. To maximize the efficiency of the vaccination campaigns, starting from 2019 ADAFSA has developed a risk-based approach for the mobilization of PPR vaccination teams relying on artificial intelligence. This approach relies on the estimation of five factors, namely: the time since the last PPR vaccination; the forecasted number of untagged animals; the detection of anomalies in terms of flock size; the proximity (<5 km) to recent PPR outbreaks; and the prediction of vaccine rejection attitudes/non-compliance. According to the conducted trials, adopting this system resulted in an improved vaccination efficiency and allowed vaccinators to reach a significant number of flocks which had been overlooked by previous vaccination campaigns. The declared goal of the vaccination program is to ensure that 80% of the SR population is immunized for three consecutive years. PVM is partially implemented, reportedly in the densest areas in terms of SR population but without covering the whole country.

Another crucial factor for PPR control is the understanding and regulation of animal movements, both internally and at national borders. This is especially true for the UAE, due to their heavy reliance on the importation of live animals to support the internal market. In particular, sheep and goats are mostly imported from Australia (only sheep), India, East Africa (Somalia) and other Middle Eastern countries (Oman, Jordan) [27]. The importance of animal trade was also confirmed by Fathelrahman et al. [100], who prepared an epidemiological model to evaluate the efficacy of different PPR eradication strategies for the UAE. Based on the obtained results, strict movement control greatly improved the efficacy of eradication efforts, in terms of reduction in outbreak duration and PPR spread, when compared to vaccination alone. Accordingly, the UAE reportedly enforce strict border security procedures, including quarantine and testing of all SRs imported from countries not officially recognized as PPR-free. Animal movement restrictions are also enacted in response to PPR outbreaks. These measures are made possible by a National Animal Identification and Registration System, which has been in place since 2010. Nonetheless, in the last PMAT update in 2020, it was declared that this system did not yet cover the entirety of the country, leaving approximately 15% of the SR population unidentified at the time and thus undermining the proper control of animal movement.

To ensure the engagement of stakeholders, regular meetings are organized by the national PPR committee using different awareness platforms, including a mobile application for farmers, which has been active at the national and local levels to allow easy access to biosecurity, treatment and vaccination guidance. Nonetheless, the participation of the private sector in the PPR control and eradication plan has reportedly been limited, especially in its early stages (2016–2019).

PPR-related activities are entirely funded through the regular governmental budget, with no external donors. Currently, the UAE is at the third stage of the stepwise approach.

### 4.15. Yemen

**Figure 10:**
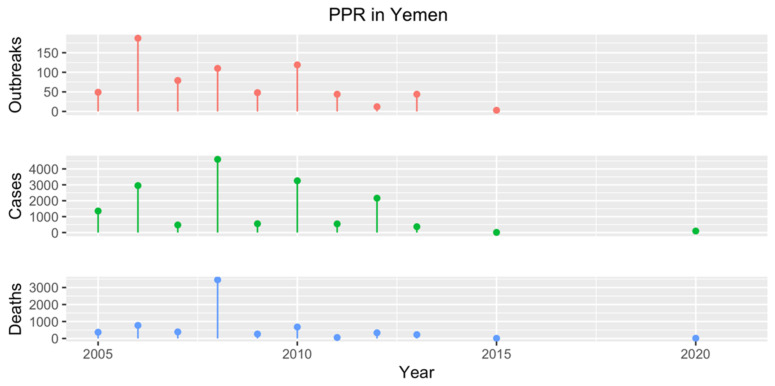
Number of PPR outbreaks (red), cases (green) and resulting deaths (blue) reported in Yemen from 2005 to 2020 according to official reports available in the WOAH WAHIS [17]. No data are available for 2014 and 2016–2019.

The first confirmed PPR outbreak in Yemen occurred in 1989 in imported sheep during quarantine in Mokha, albeit a PPR case was already suspected in 1983 in the Tihamah region. In 2000, a larger outbreak was then reported in imported Somali sheep in the Hadhramaut region and since then, PPR has been considered endemic to the country. More than 650 PPR outbreaks have been reported through the WOAH WAHIS from 2005 to 2015 [17] (Figure 10), but, after that date, its epidemiological status has become unclear due to the collapse of the surveillance system due to funding issues and shortages of diagnostic equipment and qualified personnel. Nonetheless, during a presentation at the 2022 meeting for GCC countries and Yemen, it was declared that PPR is still the leading cause of mortality among SRs in the country, highlighting the need to prioritize PPR control among SRDs.

Similarly to surveillance efforts, most PPR control and eradication activities have halted since 2015, and severe difficulties have been reported in providing even essential veterinary services. Vaccination is still conducted, but with limited coverage. In detail, 1,070,000 SRs were vaccinated in 2014 (5.6% coverage based on official SR population figures according to the FAOSTAT); 486,000 in 2015 (2.6% coverage); 39,000 in 2016 (0.2%); 27,000 in 2017 (0.1%); 2,335,000 in 2018 (13.3%); 3,502,000 in 2019 (18.2% coverage); 3,517,000 in 2020 (18.6% coverage); and 4,514,000 in 2021 (24.3% coverage) [18]. Further plans for controlling PPR were drafted in 2019 but could not be funded. Present PPR activities are entirely dependent upon external donors, such as the FAO, which supplied 6 million vaccine doses in 2020, and the International Committee of the Red Cross (ICRC), which donated over USD 5 million between 2020 and 2022. In addition, the agricultural sector as a whole has benefited from a World Bank project named “Yemen Smallholder Agricultural Productivity Enhancement Project (SAPEP)”, which amounted to USD 41.3 million and covered the period from 2017 to 2021 [101]. In light of these difficulties, Yemen is still at the first stage of the PPR GCES stepwise approach.

## 5. Discussion

To identify the main trends and constraints encountered throughout the region, we discuss below each of the five technical elements defined in the PPR GCES.

For diagnostic systems, all countries seem to possess the minimum requirements in terms of facilities and equipment to perform serology and molecular assays, except for Palestine which relies solely on RT-PCR. Sequencing, on the other hand, is reportedly available only in Egypt, the Islamic Republic of Iran, the KSA and the UAE. The actual diagnostic capacity available in the region is however hampered by several factors, including the underfunding of diagnostic services (reported by Lebanon, Palestine and Yemen), the shortages of kits and consumables (Iraq, Kuwait, Palestine, Yemen), the need for training at either the laboratory or field levels (Iraq, Jordan) and the absence of the necessary quality assurance and quality control mechanisms, including participation in proficiency tests conducted at the international level (Jordan, Kuwait).

Serious shortcomings were observed for the second technical element, which is the surveillance system. Among the most notable is the absence of official PPR reports in Qatar, despite PPRV being detected since at least 2010. The disease is reportedly still occurring in Jordan as well but has not been notified since 2000. The epidemiological situation is also unclear in Lebanon, where PPR was last recorded in 2005, in Syria, where it has not been reported in decades, and in Yemen, where the surveillance system reportedly collapsed after 2015. Other countries, such as Palestine and Kuwait, currently declare PPR as present but still suffer from underreporting. The low number of genomic sequence data from some countries, or their total absence in the case of Bahrain and Lebanon, represents another hindrance for the understanding of PPRV circulation pathways across the region. The actual impact of the disease is also poorly understood, due to the overall lack of socioeconomic studies on the subject.

Considering that PPR outbreaks have been documented in wild animals in Iraq, Iran, Israel (albeit kept in a zoo), Kuwait, Oman, Qatar, the KSA and the UAE, and in camels in Iran, the Middle East represents a good scenario for better understanding the determinants of PPR in these populations and for testing whether disease expression may depend on stress factors. The impact of PPR on wild and atypical hosts and their role in the maintenance of the disease appear, however, largely underinvestigated.

Proper surveillance relies on functioning disease reporting and animal identification systems. However, Bahrain, Kuwait, Lebanon and Palestine have reported deficiencies in disease reporting and only Qatar, Egypt and Palestine have declared having an identification system for sheep and goats, although all three countries fail to identify a proportion of their SR population. Such difficulties are largely related to nomadic farming systems still being widespread in the region, with transhumance movements which may involve multiple countries and significantly hamper the traceability of SR flocks.

Since a full comprehension of the epidemiological landscape is required to inform intervention measures, these limitations negatively impact the planning of any control and eradication activities, which represent the third technical element. Although all countries implement vaccination (expect Bahrain, which is currently preparing a dossier to apply for official freedom status), in most cases, the vaccination coverage achieved is far lower than what would be required to achieve eradication. According to PPR GCES recommendations, vaccination campaigns should be time-limited and aimed at achieving at least 80% coverage rather than being protracted but only granting limited immunization rates. After building the necessary herd immunity, in the following period, vaccination may focus only on young animals to reflect the high turnover of SR production [16]. The coverage rates reached in Egypt, Jordan, Kuwait, Oman, Palestine, Qatar, the UAE and Yemen are reportedly insufficient, while no information is available for the Islamic Republic of Iraq, Israel (where vaccination is non-mandatory), Lebanon and the KSA. The only two countries with sufficient vaccination coverage appear to be the Syrian Arab Republic (albeit only in 2020) and Iraq. However, the decrease in the number of PPR outbreaks observed in Iraq was negligible, suggesting that there may be other factors hampering the effectiveness of control efforts [63], which may include inadequate targeting of the key episystems. PVM should also be conducted to determine seroconversion rates and the efficacy of vaccination efforts. Nonetheless, Lebanon and the UAE were the only countries that declared conducting PVM activities and only during certain years or in limited areas.

Besides vaccination, the control and restriction of animal movements, both internally and across borders, represent other essential measures for limiting PPRV circulation. However, Egypt, the Islamic Republic of Iran, Iraq, Jordan, Palestine, Qatar, the KSA and the Syrian Arab Republic all mentioned difficulties in implementing them during Roadmap meetings.

The fourth technical element consists of the presence of an enabling legal framework for PPR-related activities, both at the regional and national levels. Whereas the preparation of a Regional PPR Strategy provides a solid basis for international cooperation, Palestine and the Syrian Arab Republic are the only two countries to have endorsed an NSP approved by the PPR GEP Secretariat, which is an important step towards ensuring its alignment with the PPR GCES and harmonization with other countries. Another crucial factor hampering PPR control is the lack of funding support, which was cited by Jordan, Kuwait, Lebanon, Palestine, the Syrian Arab Republic and Yemen.

The fifth and last technical element is represented by relevant stakeholders’ involvement in PPR control and eradication efforts. In many countries, the lack of awareness and compliance among farmers (and sometimes the private veterinary sector, as in Egypt, Iraq and the UAE) was reported as one of the main obstacles to disease reporting, vaccine administration and emergency responses to PPR outbreaks. Although some awareness-raising activities were conducted in a few countries, including Palestine and the UAE, this clearly highlights the need to focus on promoting participation and ensuring all the parties involved understand how PPR presents clinically, its consequences and the rationale behind control and eradication measures, thus greatly helping their enactment.

## 6. Conclusions

The PPR epidemiological situation and the status of control and eradication activities appear largely homogeneous in the Middle East, since the disease is endemic or suspected to be present in the entire region apart from Bahrain, which is also the only country where vaccination is not conducted. In addition, most countries share similar hindrances in reaching the immunization coverage needed to eliminate the disease, apart from a few exceptions. This is reflected by the low advancement in the PPR GCES stepwise approach, with 13 out of the considered 15 countries being either at Stages 1 or 2 and the other two being at Stage 3.

In this context, the highest priority action to be taken should therefore be the strengthening of surveillance systems, not limited to disease investigation and reporting, but also including the animal identification and traceability of both within-country and cross-border animal movements. This would ensure a full understanding of the local epidemiological scenario, including the role of wildlife and atypical hosts, thus informing and improving the planning and impact of vaccination efforts. There is a crucial need to identify PPR episystems as a basis for monitoring and management and to update NSPs and the Regional Strategy in line with the Blueprint for PPR GEPII, which emphasizes an episystems-led approach, enhanced access to animal health delivery services, the development of Public-Private-Community Partnerships and strengthened multi-sectoral and multi-stakeholder partnerships. The status of the Middle East as a major SR importer and exporter globally underlines the need for a clear prioritization of PPR in national strategies and livestock development planning, as well as a focus on robust import and export controls. Coordination of PPR surveillance and control activities with those of other relevant SR diseases in the region, such as sheep and goat pox, brucellosis and foot-and-mouth disease, could also be better leveraged to mutually improve their cost effectiveness.

Achieving global eradication of PPR by the target date of 2030 poses challenges, and countries and regions should engage in the risk mitigation strategies outlined in the Blueprint for PPR GEP Phases II and III [46]. For the Middle East, significant shortcomings persist, especially in relation to surveillance capabilities and conducting high-coverage vaccination campaigns for PPR. However, it should be noted that key resources required for eradication, including diagnostic laboratories and vaccine production facilities, are present in the Middle East. A crucial role will be played by the RAG and REC during PPR GEPII, and commitment and leadership at the regional level will be a prerequisite for achieving the vision of a PPR-free world by 2030.

## Figures and Tables

**Table 1:** Sheep and goat populations at the regional and national levels, according to the latest figures available from the FAOSTAT (2020).

Country	Sheep	Goats	Total
Bahrain	62,037	23,565	85,602
Egypt	29,716,608	1,067,477	30,784,085
Iran	46,587,010	16,663,721	63,250,731
Iraq	6,723,866	1,352,388	8,076,254
Israel	500,000	116,293	616,293
Jordan	2,969,798	661,266	3,631,064
Kuwait	725,697	214,529	940,226
Lebanon	431,718	534,497	966,215
Oman	629,742	2,395,243	3,024,985
Palestine	766,546	213,826	980,372
Qatar	1,021,718	458,835	1,480,553
Saudi Arabia	9,446,699	6,100,000	15,546,699
Syrian Arab Republic	16,073,088	1,995,923	18,069,011
United Arab Emirates	2,006,069	2,378,168	4,384,237
Yemen	9,238,763	9,678,845	18,917,608
TOTAL	117,660,596	43,854,576	161,515,172

**Table 2:** Self-reported numbers of small ruminants vaccinated yearly against PPR in Jordan and respective coverage rates.

Year	Vaccinated Small Ruminants	Coverage
2016	1,837,323	40%
2017	1,623,142	36%
2018	1,024,044	22%
2019	1,285,326	30%
2020	1,596,616	35%
2021	1,665,138	Unknown

**Table 3:** Number of small ruminants vaccinated yearly against PPR in Qatar, as presented by national representatives during the 2022 meeting for GCC countries and Yemen. The respective coverage rates, calculated based on official population figures from the FAOSTAT [18], are also provided.

Year	Vaccinated Small Ruminants		Coverage	
	Sheep	Goats	Sheep	Goats
2017	156,150	22,050	16.7%	5.8%
2018	162,743	28,930	16.3%	7.1%
2019	170,510	30,941	16.9%	7.0%
2020	72,601	22,960	7.1%	5.0%
2021	346,427	66,210	Unknown	

## Data Availability

Not applicable.

## Supplementary Materials

The following supporting information can be downloaded at: https://www.mdpi.com/article/10.3390/ani13071196/s1, Figure S1: Map of the region showing the distribution of sheep adjusted to FAOSTAT population figures for 2020, with SR density (head per square km) indicated by the colored shading; Figure S2: Map of the region showing the distribution of goats adjusted to FAOSTAT population figures for 2020, with SR density (head per square km) indicated by the colored shading; Figure S3: Share of global domestic SR (sheep and goats) imports and exports attributed to countries included in the present study, according to data gathered in 2020 by the Observatory of Economic Complexity [27]; Table S1: Placement of Middle Eastern countries within the stepwise approach of the PPR GCES according to the latest self-assessment data.
