# Supplementary material for: Peste Des Petits Ruminants in the Middle East: Epidemiological Situation and Status of Control and Eradication Activities after the First Phase of the PPR Global Eradication Program (2017–2021)
Source: Animals (Basel). 2023 Mar 29;13(7):1196. doi: 10.3390/ani13071196 (PMC10093352; doi:10.3390/ani13071196)
Supplement: Supplementary file 1 [file animals-13-01196-s001.zip › animals-2276089-supplementary.pdf]

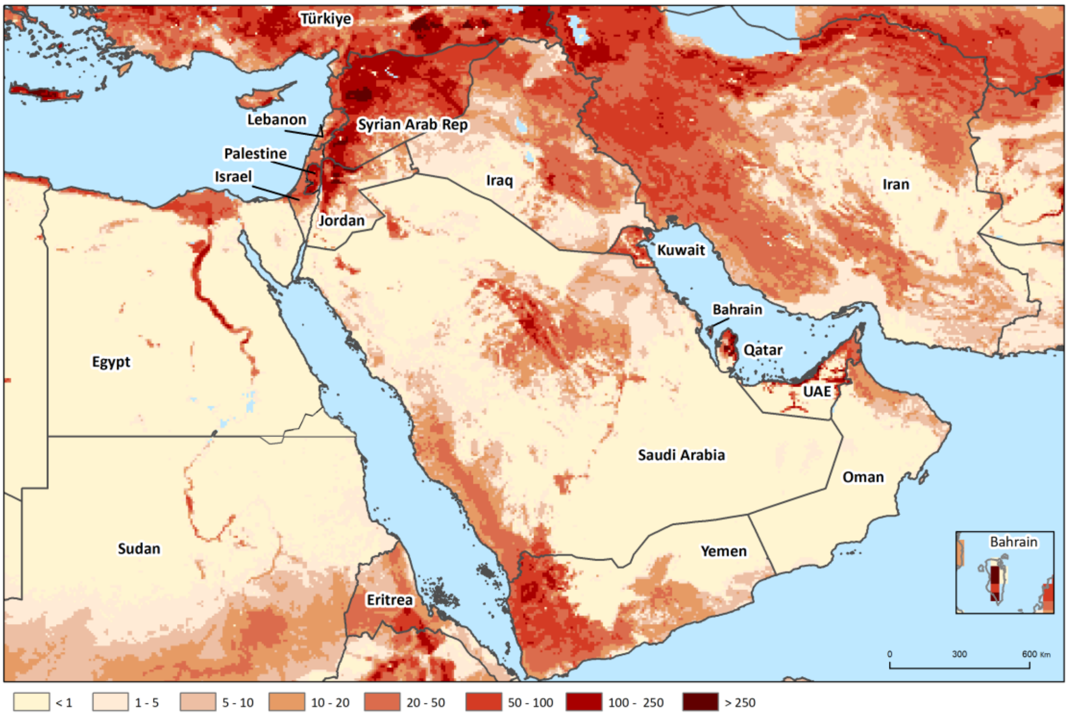

Source: **United Nations Geospatial.** 2020. *Map of the World. United Nations.* Cited 22 August 2022. www.un.org/geospatial/file/3420/download?token=TUP4yDmF modified with GLW4 2020 data.

Figure S1. Map of the region showing the distribution of sheep adjusted to FAOSTAT population figures for 2020, with SR density (head per square km) indicated by the colored shading;.

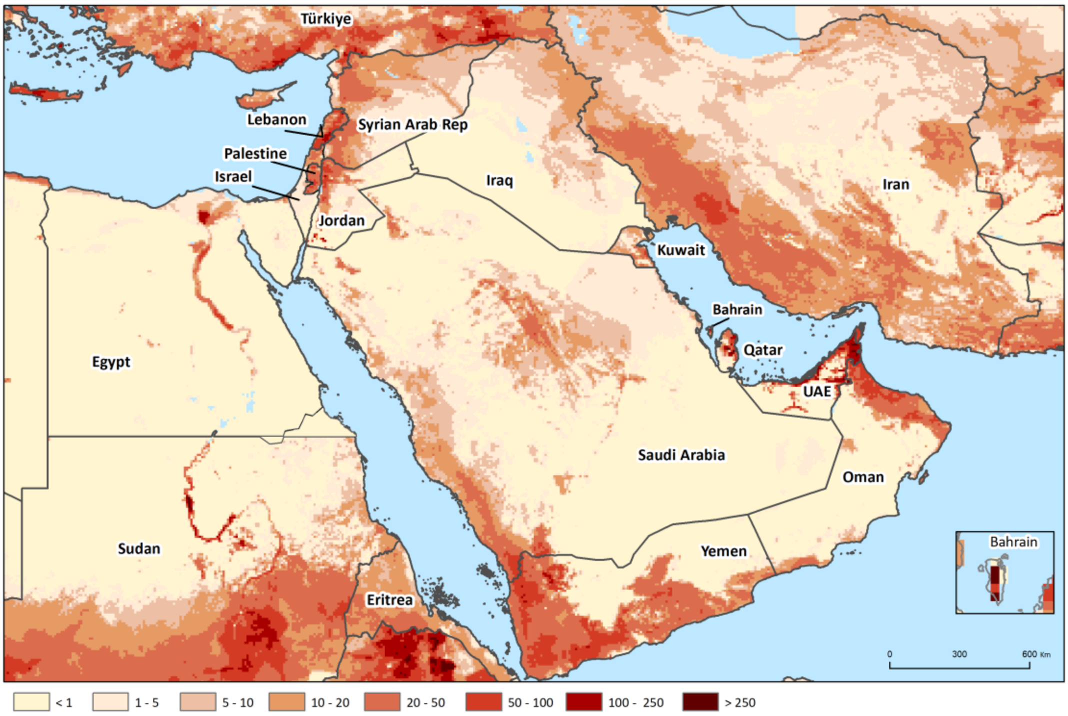

Source: **United Nations Geospatial.** 2020. *Map of the World. United Nations.* Cited 22 August 2022. www.un.org/geospatial/file/3420/download?token=TUP4yDmF modified with GLW4 2020 data.

Figure S2. Map of the region showing the distribution of goats adjusted to FAOSTAT population figures for 2020, with SR density (head per square km) indicated by the colored shading;.

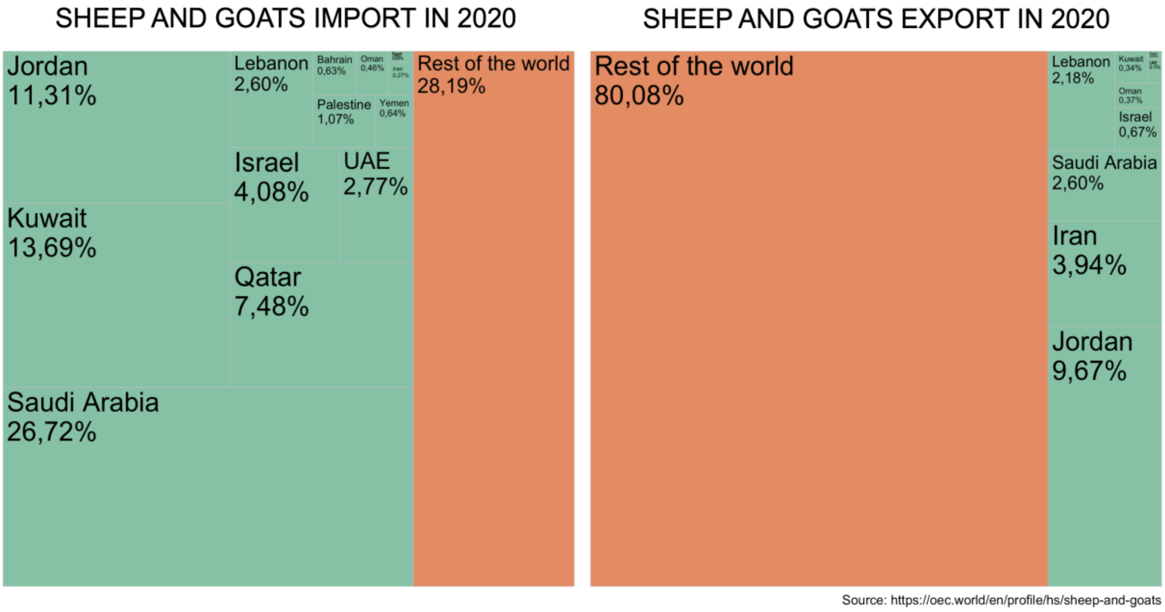

Figure S3. Share of global domestic SR (sheep and goats) imports and exports attributed to countries included in the present study, according to data gathered in 2020 by the Observatory of Economic Complexity [27];.

Table S1. Placement of Middle Eastern countries within the stepwise approach of the PPR GCES according to the latest self-assessment data.

Country	Last update	Stage 1<br>Assessment stage	Stage 2<br>Control Stage	Stage 3<br>Eradication stage	Stage 4<br>Post eradication stage
Bahrain	2022				
Egypt	2021				
Iran	2021				
Iraq	2017				
Israel	2021				
Jordan	2022				
Kuwait	2021				
Lebanon	2021				
Oman	2022				
Palestine	2022				
Qatar	2022				
Saudi Arabia	2022				
Syrian Arab Republic	2021				
United Arab Emirates	2022				
Yemen	2022				
